# Domestication as Stabilisation: Exploring the Incorporation of Social Technology by Older Adults and Their Relatives

**DOI:** 10.1111/1467-9566.70131

**Published:** 2025-12-02

**Authors:** Martin Vinther Bavngaard, Anne Lund, Erik Børve Rasmussen

**Affiliations:** ^1^ Department of Rehabilitation Science and Health Technology Faculty of Health Sciences Oslo Metropolitan University Oslo Norway; ^2^ Department of Social Work, Child Welfare and Social Policy Faculty of Social Sciences Oslo Metropolitan University Oslo Norway

## Abstract

As health and care policies in Western societies emphasise ageing at home, loneliness among home‐dwelling older adults emerges as a health‐related area of intervention and technological innovation. One such intervention is Komp, a social technology designed for families to socially include an older family member struggling to use digital technology. This study couples the concepts of domestication and script to unpack the negotiations of Norwegian older adults and their relatives during Komp's introduction into their sociotechnical constellations. Utilising a multimethod longitudinal design, our inquiry produced 46 interviews, 22 photos and accompanying participant observations. From this empirical foundation, a reflexive thematic analysis was undertaken to develop our understanding of domestication trajectories. Our analysis foregrounds *stability*, *volatility* and *breakdown* as dynamic states involving different degrees of harmony in the negotiated meaning and use of Komp among a constellation of actors. Beyond a novel rendition of domestication as states of (in)stability, this study encourages an understanding of the effects of technological interventions as always in production, sustained by users' continuous negotiations involving the technology's scripted design, other technologies and—importantly—themselves.

## 1 Introduction

Facing unprecedented rates of population ageing, Western societies have oriented health and social policies towards ageing‐in‐place to reduce healthcare costs by postponing resource‐intensive residential care (The United Nations Economic Commission 2022). Loneliness among home‐dwelling older adults constitutes a risk factor for residential care admission (Hanratty et al. 2018), and the projected doubling of lonely older adults in Norway by the year 2050 has cast loneliness as a subject for national economic and health policy initiatives (Aunsmo et al. 2023; Meld. St. 24 2022–2023). As noted by Monteiro et al. (2024), retirement, loss of family and friends, reduced mobility and chronic disease can reduce older adults' social contact frequency (i.e., social isolation), potentially fostering a discrepancy between wished‐for and actual social connection (i.e., loneliness). Chronic disease, in particular, may impede intentions to counteract isolation, producing a downwards spiral of loneliness (Wotherspoon 2024). Beyond the conventional understanding above, loneliness lacks an agreed‐upon conceptualisation. Its status as a health risk, however, cuts across its epistemological multifariousness and have cultivated efforts to render it—and, importantly, the effectiveness of interventions against it—quantifiable (Malli et al. 2023). Simultaneously, reducing loneliness constitutes an emerging site for technological innovation, as recent interventions are increasingly digital (Fakoya et al. 2020).

**FIGURE 1:**
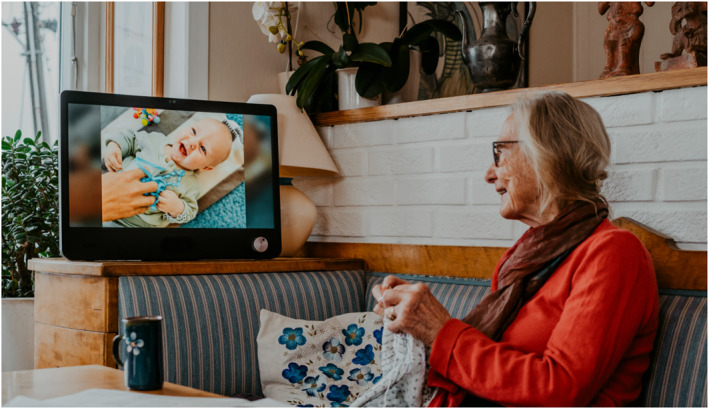
Komp's picture display. Photo by Esra Johnsrud for Abilia.

This study explores the case of one such intervention: the social technology Komp. Social technologies are technologies designed to connect individuals for social purposes (Wilson et al. 2023). Developed by the Norwegian start‐up *No Isolation* (now acquired by *Abilia*), Komp is intended to combat loneliness among older adults struggling to use digital technology. Low technical competence remains well established as a barrier for older adults' use of technology to achieve social connectedness (Wilson et al. 2023). To circumvent this barrier, Komp represents a *gerontechnology*: technology designed for—and, in Komp's case, in collaboration with—older adults (Peine et al. 2022, 2; Brænden et al. 2018). The outcome of this co‐design process is a dual‐artefact solution comprising a 21‐inch screen, stationed in the older adults' home, and a smartphone app through which relatives can send messages and pictures and initiate video calls (Figure 1). Although messages and pictures represent asynchronous communication modes, curating content meant to be viewed at the recipient's leisure, video calls initiate a ten‐second countdown displayed on both the app and the screen, after which a synchronous audiovisual feed is established. The video call request is automatically answered and can only be declined by the recipient turning off the Komp screen. Its single button, once turned, will switch the screen on/off, with additional turning increasing volume levels. Thus, the Komp screen user cannot initiate contact with the app users—only vice versa. Komp, then, is intended as a communication hub relieving the older adult of technical competency requirements by shifting the responsibilities of initiating contact onto app users.

Although previous research points to an overall satisfaction with Komp (e.g., Akhtar 2024), less has been written on the prescriptiveness of its design, including its implications for attaining the envisioned effect on loneliness when taken into use. Technologies do not merely ‘work or fail in and of themselves’ (Mol et al. 2010, 14) and neither does Komp. Rather, users' attempts at incorporating technology give rise to a contested space comprising ‘designers' views and users' needs and interests’ (Sørensen 2006, 46). Alarm pendants may reproduce specific notions of successful ageing (Aceros et al. 2015), medicine dispensers reconfigure pre‐existing care arrangements (Kleiven et al. 2020), and robot bathtubs jeopardise feelings of dignity (Beedholm et al. 2016)—all consequences not accounted for by their framing as interventions towards specific issues. The *interpretative flexibility* (Bijker and Law 1994; Orlikowski 1991) or *multistability* (Verbeek 2005) of technologies implies that understanding their supposed effects involves an exploration of users' efforts to manage their openness through sociotechnical practices. To investigate how older adults and their relatives contend with the predesigned openness of Komp, we couple the concepts of *domestication* and *script*. In doing so, we expand the understanding of both the empirical subject of our inquiry (social technology) and the enquiry's modus operandi (domestication) by showcasing the centrality of *stability‐in‐constellations*.

### 1.1 Theoretical Framework

In its metaphorical rendition of a technological artefact as a beast to be tamed, the domestication framework emphasises the complex processual dynamics of negotiating technology's roles in the lives of its users. Informed by two complementary schools of thought, we regard the dynamics of domestication as distinct but interwoven processes of appropriation, objectification, incorporation and conversion (Silverstone and Hirsch 1994) and involving practical, symbolic and cognitive dimensions (Sørensen 2006). This latter school's conceptualisation of domestication served, in part, to move attention outside the original household setting, emphasising *what is being domesticated* while downplaying *the domestic*. This study returns to the domestic but takes domestication to entail multisited user negotiations (Johannessen et al. 2023); as previously mentioned, Komp comprises dual artefacts, with only one situated within the boundaries of home. Drawing on theoretical tenets from science and technology studies, we develop Sørensen's (2006) rendition of domestication as the introduction of artefacts in networks. With its explicit reliance on networked interconnectedness, the case of Komp illustrates how the abovementioned processes and dimensions do not merely push actors along a linear axis between successful and failed domestication but land them in dynamic states marked by *stability*, *volatility* or *breakdown*.

The attention to users' practices emphasised by the domestication framework does not render technologies empty signifiers with unbounded flexibility, wholly malleable to their users' intent. The concept of *script* denotes how technologies bear marks of their production by embodying designers' ‘beliefs about the relationship between an object and its surrounding actors’ (Akrich 1994, 208). Relevant to our analysis, Rasmussen et al. (2021) identified numerous aspects of Komp's scripted design, notably (1) relatives as intended app users, (2) assumed technical competence among relatives, (3) asymmetrical roles between the passive older user who consumes content produced by active relatives and (4) one particularly close relative to be delegated the administrator role. Drawing upon these four aspects, we demonstrate how a core component of domesticating Komp is for users to contend with its scripted nature by adhering to it, resisting it or attempting to rescript it (Akrich 1994). Although it does not strip users of their agential power, Komp's script presents an imperative of active negotiation (Oudshoorn and Pinch 2003) which influences the stability of their collective domestication. As an analytical concept, script encompasses rejection and nonuse in addition to different types of use.

Despite enjoying mutual scholarly acknowledgement (see Silverstone and Hirsch 1994; Sørensen 2006), domestication and script have been coupled by relatively few empirical studies (cf. Kleiven et al. 2020; Aceros et al. 2015). Together, the concepts accentuate the interplay between Komp's prescriptive design vis‐à‐vis users' practices of incorporation across different sites, affording an understanding of Komp's domestication that moves beyond a binary matter of ‘success‐or‐not’ to one of stabilising constellations of human and nonhuman actors.

## 2 Materials and Methods

### 2.1 Materials

Although its parent project BoVEL employs a randomised controlled trial (RCT) to evaluate Komp's effect on older adults' loneliness and, consequently, their ability and inclination towards ageing‐in‐place, this qualitative study explores the processual dynamics shaping any such effect. In collaboration with homecare services in three city districts of Oslo, Norway, 300 Komp screens were distributed among older adults, free of charge, by project coordinators in each district. Working within the services, these coordinators acted as proxies for recruiting informants by suggesting study participation to families during home visits. Because recruitment adhered to the concurrent RCT's design, the coordinators applied criteria for inviting older adults to participate, dictating that these be (1) above the age of 67, the normative retirement age in Norway; (2) living at home in one of the three city districts; (3) living with a moderate degree of functional impairment and (4) granted community‐based homecare services. Once oral consent had been provided by potential informants, their contact details were handed over to the research team for a formal invite. Additionally, researchers joined project coordinators during their home visits, personally extending the invitation to participate.

### 2.2 Data Production

To investigate domestication trajectories over time, the study employed a longitudinal design with data production spanning from February to November 2023 and involving multiple modes of inquiry, all enacted by the first author. Individual interviews were conducted 1 month after initially acquiring Komp with the older adult and one close relative (or friend)—often the assigned administrator of the Komp app. These informant pairs, we refer to as *dyads*. After a subsequent period of no less than 3 months, a second interview was conducted with each dyad. Both interviews were semi‐structured and framed by an interview guide which maintained flexibility to pursue relevant themes not captured by prespecified questions. Prior to the second interviews, the interview guide was tailored to each informant to include themes that surfaced in the first interview. As per our theoretical anchoring, the interview guides encouraged accounts on dyads' entire Komp network in addition to their own use. Although interviews with all but one relative were conducted via a secure Zoom connection to offer flexibility, interviews with the older adults took place physically in their home. Choosing this site for interviewing allowed for (1) the informant to feel more at ease, (2) the dialogue to take place in physical proximity to the Komp screen, (3) an impression of the home environment and (4) the researcher to capture photos. Although the first two points reference methodical techniques for stimulating reflection and articulation (Pain 2012), proving particularly fruitful for some older informants, the third point represents a data production method, as impressions of the home and nonverbal aspects of the interview itself were described in reflexive notes after almost all interviews. Photos were taken of Komp's placement with two purposes: first, as data depicting the spatial aspects of domestication from a theoretical conviction of the ‘importance of place’ (Oudshoorn 2012, 125); and second, as a method of elicitation by presenting the photo from the initial interview during the second (Quinton et al. 2023). Lastly, participant observations conducted during project coordinators' handovers of Komp often captured the initial moments of dyads' acquisition and installation, thereby offering contextual insights supplementing the interviews and photos.

Altogether, this study draws on 46 interviews with 12 dyads encompassing older adults and relatives, 22 photos of Komp's placement and observations during eight Komp handover visits. Interviews with older adults totalled 19 h and 36 min, with the average interview lasting 52 min; for relatives, the total interview data comprised 18 h and 25 min, with each interview averaging 48 min. Reported age ranged from 72 to 96 for the older adults (mean age 84) and from 28 to 63 for the relatives (mean age 53). Dyads predominantly comprised women, with 10 female and two male older users and a corresponding distribution among relatives. Although most relatives lived outside Oslo, older adults dwelt in the three city districts in a six‐three‐three division. Apart from one dyad, who returned Komp after 49 days, all dyads had kept Komp between five and 8 months by the time of the second interview.

### 2.3 Data Analysis

All interviews were audio recorded and transcribed verbatim in Norwegian by the first author, following prespecified transcription conventions and using the qualitative analysis software NVivo R1. Informant names were pseudonymised to ensure confidentiality during transcription. Our analysis leveraged the flexibility of Braun and Clarke's (2022) six‐stage reflexive thematic analysis. This method allows for theoretically driven analyses comprising heterogeneous dataset compositions, whereas its emphasis on conveying stories through the ‘patterning of meaning across the dataset’ (Braun and Clarke 2022, 76) aligned well with our longitudinal design and focus on domestication trajectories.

Analysis began with the decision to retain the preceding dyadic structure of the data, each dyad comprising four interviews and two photos (save for the dyad who returned Komp). Driving this decision was our theoretical emphasis on multisited negotiations and our intention for the analysis to move beyond each dyad's domestication trajectory to address patterns concerning the totality of the dataset, as we sought to explore how domestication unfolded across dyads. This meant treating dyads as our principal units of analysis. In line with this decision, familiarisation involved constructing narratives for each dyad detailing both the first and second interviews while noting all relevant details regarding informants' attempts at incorporating Komp. These narratives constituted a 35‐page document for (re)tracing the overall dynamics interpreted from the data during the subsequent coding process. Each dyad was coded individually by the first author, operationalising Sørensen's (2006) and Silverstone and Hirsch's (1994) domestication concepts and Akrich's (1994) notion of script. Codes captured both surface‐level and conceptual meaning units, and although some depicted isolated instances, others acted as broader categories comprising several instances without sensitivity to the particular way in which each instance manifested (Braun and Clarke 2022). Across all dyads, the first author developed a total of 780 codes. During this coding process, and integrating the domestication and script concepts, three preliminary themes were conceptualised. Roundtable discussions involving all authors served to develop the three themes' conceptual delineations by collaboratively distributing printed‐out snippets of all codes among the themes, revealing cases of misfit. In refining the themes, the first author revisited both the codes and the initial narratives for each of the dyads to combat undue decontextualisation. Approaching the data from this less abstracted position (Braun and Clarke 2022, 101) helped establish the three conceptually mature themes presented below as empirically grounded abductive constructions.

### 2.4 Ethics

As emphasised by Nordtug and Haldar (2023), an ethical study must venture beyond institutional approvals to tackle issues emerging during its undertaking. To complement the recommendation by the Norwegian Agency for Shared Services in Education and Research (no. 589119) and approval by the Regional Committees for Medical and Health Research Ethics (no. 516796), we therefore comment on salient ethical issues arising during our study's progression. As Komp is designed to also be usable by people living with dementia, our study specifically sought to include these as informants. Although none were deemed ineligible to consent by the recruiting project coordinators (all healthcare professionals), four of our informants lived with dementia in varying stages of progression. All four personally signed their consent form, but upon returning to conduct the interviews, some greeted the researcher as either a technician or the inventor of Komp, calling into question whether their consent could truly be deemed ‘fully informed’. In attempting to practise ethical consideration while avoiding naïve paternalism, we maintained that interviewing older people with various impairments should involve strategies sympathetic to their individual capacities (Amoss et al. 2011, 4). These included inviting the close relative to sit in on interviews to provide support, as well as sharing the interview guide with the informants to collaboratively formulate the questions.

## 3 Results

Our three themes represent three distinct trajectories for Komp's domestication by families, showcasing how these either settle into a state of *stability*, *volatility* or *breakdown*. These states are viewed not as absolute endpoints but as dynamic, holding intrinsic, albeit varying, capacities for change. Each theme foregrounds specific dyads' domestication trajectories as examples illustrating intratheme variations. Table 1 provides an overview to accompany our narrative, depicting the state of all 12 dyads' domestication at the time of the final interviews.

**TABLE 1:** Overview of domestication trajectories across all dyads.

Informant pairs as dyads	State of stability	State of volatility	State of breakdown
1	X		
2	X		
3	X		
4	X		
5		X	
6	X		
7			X
8		X	
9	X		
10		X	
11		X	
12			X

### 3.1 Stability: Komp as Temporarily Tamed

Establishing stability denotes that the meaning and use of a technology have synergistically reached a (temporary) closure due to actors' negotiations involved in their domestication efforts. As shown in Table 1, six of the 12 dyads had achieved a state of stability by the time of the follow‐up interview, although getting there involved disparate practices. To unpack these, we first highlight Dyad 6, whose 5‐month long domestication of Komp involved accepting its script. Symptomatic of all six dyads achieving stability, Komp's acquisition was significantly influenced by relatives. Indeed, Ella (in her late fifties) had convinced her mother, Maude (early nineties), to accept the offer to receive Komp despite Maude's worries that it might not only be too technical but also supplant physical visits. Reassured that Komp would claim no visits, Maude had let the technology cross the doorstep. Rendering Komp an object of desire, however, was still an ongoing process after the first month. Although Ella added several family members who actively engaged with Komp's picture and video call functions, Maude remained less eager to appropriate Komp. Taking an acquiescent stance towards her daughter's enthusiasm, she maintained that Komp presented ‘too much newness’ and that it held neither practical nor symbolic value to her. Viewed from the cognitive dimension of domestication (Sørensen 2006), Komp's entry into Maude's family had painted to her as a wild stranger—in part due to her advanced dementia condition. Correspondingly, domesticating Komp was no trivial matter, as appropriation and incorporation implied comprehending Komp and its logic. This required persistent efforts from Ella: reinserting power cords pulled out by Maude; drawing pictures displayed on the screen into casual conversations; shifting the mode of contact from Maude's Doro phone (an easy‐to‐use senior phone much more familiar to her) to Komp and directing her to talk at Komp rather than into her phone during video calls. Moreover, Ella would sometimes have to call her mother on her phone, instructing her to move from the kitchen towards Komp stationed in the living room prior to initiating a video call. To further aid Maude's comprehension, Ella added descriptive captions to all pictures and shielded her mother from Komp's message function, acknowledging that Maude did not yet understand that she must routinely look at the screen for this to be a useful function. For Ella, incorporating Komp thus involved constructing practices to establish its meaning and use for Maude to encourage its transformation from stranger to friend. As illustrated by the examples above, this included counteracting behaviour by her mother discordant with Ella's interpretation of Komp's intended use. By adhering to Komp's script, wherein technically competent relatives take on the brunt of the incorporation work, Ella's efforts established a reciprocity of practices conducive to stable use. At the 5‐month mark, incorporation had gone beyond the stage of comprehension:

Interviewer Have you gotten used to [Komp]?

Maude Yes. It's standing over there in the corner, talking.

Indeed, Maude no longer mistook Komp for the phone ringing and knew to move to the living room once she heard its chime. As a final attest to its domestication achieving a state of stability, Komp's power cord was now left plugged in—a salient difference from Maude's digital clock, which had suffered a different domestication trajectory. Through ongoing negotiations, the dyad had established meanings proving to be synergetic: Although Maude enjoyed viewing pictures and experienced video calls as more intimate compared to phone calls, her daughter found great reassurance in video calling to check on Maude's condition, as she would not activate her safety alarm in case of a fall incident. Although nonaligned, the actors' established meanings were harmonious, allowing for stability in their joint attempt at domestication. Moreover, as Maude increasingly struggled to operate her phone, sometimes turning it off, muting it or forgetting how to answer it, Komp fulfilled an emerging practical need for both actors by sustaining a line of communication. Altogether, established meanings and needs co‐existed to stabilise the constellation surrounding Komp during its domestication.

Contrasting Dyad 6, the path of Dyad 9 towards stability entailed rescripting Komp as part of their domestication. Both Doris (early nineties) and her granddaughter, Sophie (late twenties), welcomed Komp as a communication solution, having failed consistently in using FaceTime. As in Dyad 6, a need to establish a sustainable communication pathway had emerged, accentuating Komp's potential. Sharing the role of Doris' caregiver with her mother, Sophie mirrored their close‐knit relationship on Komp and was quick to engage with its picture and video call function. As part of the negotiations in incorporating Komp, Sophie had increased her activity to meet her grandmother's expectations:

Grandma thought […] that there was not enough action. Because she‐ I kinda posted a picture… I don’t know, maybe once a week or something like that, and then‐ then she saw no point of having it on.

Adopting the familiar logic of short‐format Instagram Reels, Sophie now posted new pictures every day, with most of them expiring after only a couple of days. Ipso facto, she had established a new precedent for what constituted stable use, shaping Doris' expectation of content provision. This stability, however, also introduced disharmony in Komp's established meaning across the dyad: Despite Sophie considering her efforts ‘tiring’ and ‘unsustainable’, frequent posting was necessary to alleviate her grandmother's feeling of loneliness and to keep Komp switched on. Sophie's acknowledgement that the enrolment of additional members would offer relief gave way to her worry that their entry into the close‐knit network would reconfigure it, leading to a loss of intimacy between Sophie and Doris, but also between Sophie and her mother, who both used the app to stay involved in each other's lives through their respective posts. By taking upon herself a responsibility designed to be distributed across a larger family network, Sophie's use diverged from Komp's script. This divergence allowed her to retain the close‐knit constellation, but at the price of sustaining disharmonious practices and meanings across the network, thereby challenging a stable domestication. During the following 6 months, Sophie gradually sought to rectify this divergence due to its unsustainability by inviting additional members. When none accepted, she doubled down on rescripting Komp to fit the tripartite network by tackling the disharmony surrounding Komp's use head‐on. Verbally renegotiating its established meaning with her grandmother eventually led to Doris' ‘hesitant acceptance’ of Sophie's less intensive but more sustainable use. No longer a content funnel demanding unsustainable effort, the rescription allowed for a stable alignment of Doris' and Sophie's reciprocal derivation of value through Komp's use.

Although both dyads' domestication trajectories achieved a state of stability, such closure was anything but permanent. Discordance could be (re)introduced into the sociotechnical constellation by the dynamic context enveloping each dyad: For example, the prospect of homecare services joining Komp to coordinate home visits excited Ella, whereas her mother, Maude, remained indifferent. Similarly, on the day of the last interview with Doris, a family member suddenly connected, thus expanding the close‐knit network. Such dynamics bring about the opportunity and—sometimes—need for renegotiation; indeed, Doris announced that she looked forward to *more* activity on Komp due to the new member's arrival.

### 3.2 Volatility: Komp as Docile‐But‐Feral

**FIGURE 2:**
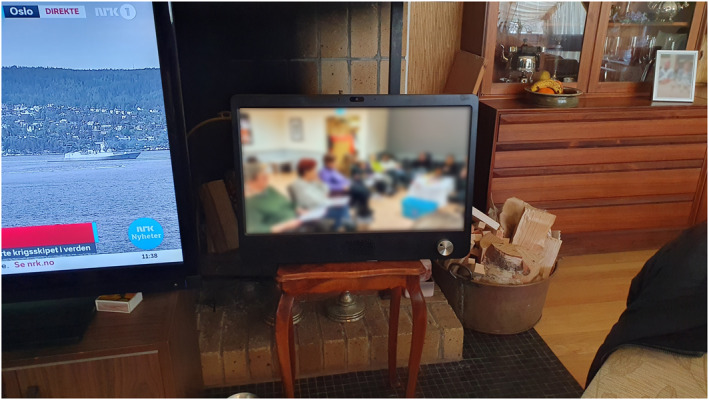
Komp's placement in Henry's home.

A state of volatility signifies that actors' negotiations have not resulted in them establishing a stable meaning and use. As absolute alignment between views on Komp's meaning is not a prerequisite for stability, neither is absolute nonalignment for volatility. Different views can, variously, be in harmony or disharmony, with volatility a product of the latter. In such cases, the constellation surrounding Komp remains fickle, marked by unresolved tensions across actors and sites. Four dyads inhabited this state, with volatility manifesting differently for each. Although dyad 9's rescripting of Komp paved the way for stability, Dyad 5's attempt at rescription created volatility. A frail man in his mid‐nineties, Henry spent most of his day in his favourite chair and received daily care from both his wife and homecare services. When acquired, Komp was quickly cast as desirable to Henry, his wife and his son Lars (early fifties). Many incorporative practices instrumental in establishing stability were undertaken: Komp found a physical placement on its own stool next to the TV (Figure 2), viewable from Henry's armchair and framed to allow callers to see him; three of the four connected family members frequently posted pictures; and Henry described video calls as feeling intimate, ‘like there's two persons in the room. There's not one, but two’. Komp's scripting as a social technology was enacted in these practices.

However, being the principal users (and administrators), Henry's wife and son gradually negotiated a use for Komp diverging from sociality. Despite Henry disclosing feeling lonely and that Komp alleviated such feelings, Lars maintained that his father was not lonely. Rather, Komp became constructed as a tool for safety. As Henry could no longer operate his Doro phone, his only option for contacting others when alone was his safety alarm. Thus, Komp's video call function served as a means for his wife and son to regularly assess his condition, including whether he had taken his medication or eaten the food prepared for him. This use allowed Henry's wife to venture outside the home for longer than a few hours and absolved Lars of the need to travel to his father—enabling a coordination of caregiving tasks across sites and, by extension, reconfiguring Komp's meaning to inhabit a more instrumental quality:

I called him on Komp when I was gone for some hours this morning. Then I was able to see that he sat in his chair and how he felt. And [I] saw that he hadn’t touched his food, and then I told him ‘you’ve got to eat your food and empty that glass'

(Henry’s wife)

Used to his role as patient, Henry accepted his wife and son's use. However, rescripting Komp as a tool for safety cast its design as less than optimal for fulfilling the newly established needs. Difficulty in capturing his father's attention when dozing or watching television in his chair led Lars to desire more invasive functions, such as integrating Komp with existing sound systems to mute the television and radio when video calling. Likewise, and despite Henry's own indifference, Lars proposed that Komp be connected to an emergency button, enabling him to call others if in need. Rescripting Komp also introduced conflicting values, as Komp's placement next to the television was necessary to both capture Henry's attention and assess whether he had finished his food, drinks and medication—despite him repeatedly confusing content on Komp and the television. We argue here that the rescription of Komp left something to be desired for Henry, his wife and his son and that this disharmony introduced volatility. Alongside this rescripting, interfamily dynamics challenged stability, from tensions between Lars and his daughter leading to demonstrative nonuse to the exclusion of Henry's cognitively impaired daughter due to her inability to use the Komp app and his inability to operate his Doro phone to accept her calls.

**FIGURE 3:**
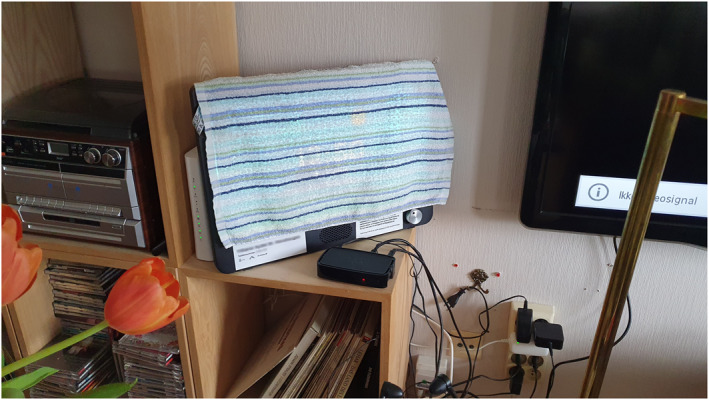
Komp's primary mode of existence in Anna's home.

Although the case above illustrates how volatility can shape use rather than inhibit it altogether, the following case exemplifies this latter outcome. Despite deciphering from the informational material that she was not a conventional user, having no family or children, Anna (early eighties) nonetheless wished to receive Komp. Her constructed imaginary cast Komp as ‘an open door’ for her friends to contact her. However, swapping family for friends constituted a script divergence influencing domestication from the get‐go. Because Komp's script demanded the administrative role be given during setup, the local project coordinator appointed this role to Anna's friend Lotte (mid‐seventies). Because of (1) close relatives being intended keepers of this role and (2) the assumption of high technical competency among connecting relatives, both embedded in Komp's script, this initial misfit came to significantly inhibit incorporation. Although Lotte accepted her role, the project coordinator's enaction of Komp's script frustrated Anna, who disliked being put in a role of passivity and preferred that the responsibility of inviting members be placed upon her rather than her friend. Sceptical of Anna's technical competency, Lotte herself admitted that adhering to Komp's script had been difficult: Beyond struggling to set up her own app, she had failed to guide potential members through the setup procedure. Despite Lotte's persistent efforts, the incorporation of Komp had, in the words of Anna, ‘kind of just ran into a wall’. At the 6‐month mark, Komp displayed only a single picture taken by the first author during Lotte's initial interview to demonstrate content posting. Likewise, the number of video calls could be counted on one hand. Outside of video calls being ‘cosy’, Komp had obtained no significant value to Anna, and she had contemplated returning it. Convincing her not to, Lotte admitted that Komp might hold more practical and symbolic value to her than to Anna, attesting to the discrepancy in its established meaning across the two friends. Komp's inactive presence in Anna's home had gradually demoted it from its status as a potential communication hub to little more than a flashing screen. Indeed, at the last interview, she felt that ‘it's used so little, so it's just kind of a piece of furniture’. As an ultimate sign of its loss of meaning, Figure 3 illustrates how Anna would cover Komp with a washcloth, as its single‐picture display was deemed disturbing while reading and watching TV. Starting their domestication of Komp from a position of script nonconformity, Dyad 10 had entered a fickle state, marked by volatility.

### 3.3 Breakdown: Komp as Remaining Wild

**FIGURE 4:**
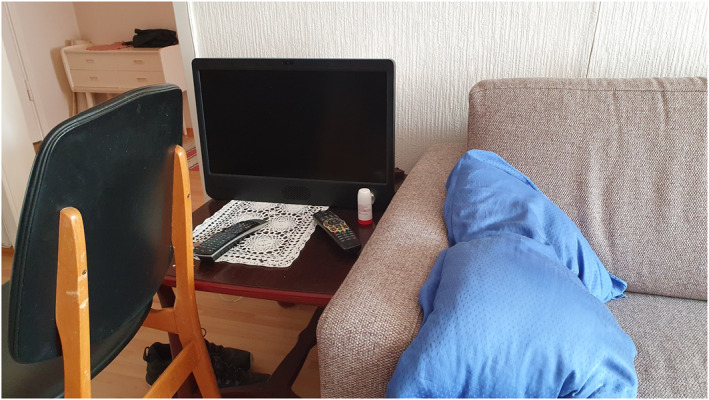
Haakon's Komp, turned off.

Contrasting stability, the state of breakdown denotes that actors' negotiations during domestication are marked by either (1) a sustained presence of incompatible views on Komp inhibiting the establishment of a stable meaning and use or (2) an outright rejection of the technology by key actors. Departing from the former scenario, we consider what characterises breakdown vis‐à‐vis the volatility exemplified above by Dyad 10. Assuming that *appropriating* a technology involves negotiating its degree of desirability (Johannessen et al. 2023, 4), the negotiations initiated by dyad seven largely remained within this process. For Haakon, a man in his early nineties, Komp was overwhelmingly an object of nondesire. Inherently sceptical of new technology, effort was required from both his daughter, Berit (early sixties), and the project coordinator to convince him to agree to a 2‐month trial run. Repeatedly insisting not to let his daughter make any decisions on his part, Haakon's relationship to Berit seemed marred by tensions. Being ‘both close and not so close’ to her father, Berit initially sought to rescript Komp from a social tool to an information delivery tool to fulfil her own needs as his caregiver. For this, Komp held great value, as it allowed her to display text messages to inform and remind her father of various appointments, circumventing the need for her to travel. Importantly, it absolved her from making phone calls, which often led to misunderstandings when coordinating care due to Haakon being very hard of hearing. However, although Berit had successfully appropriated Komp to suit her needs and had begun incorporating its functions into her everyday life, Haakon still viewed Komp as an unwelcome character. After 1 month, he reported no effect: He did not feel more socially connected to his two daughters comprising the Komp network, and although he found video calls to facilitate better conversations due to their visuality, he derived ‘no use of the screen at all… It's just no use’. This, in conjunction with his dissatisfaction at not being able to initiate calls via Komp, had led Haakon to increasingly keep the technology turned off, rendering it simply a black screen in his living room (Figure 4).

Like Anna's covering in Dyad 10, keeping Komp turned off served to question its status as a communication hub. Haakon's questioning notwithstanding, Komp was still in use: Berit had learnt to routinely check Komp's status indicator in the app to coordinate her posting practices and ensure that messages were received. On her end, she was still able to derive value from its established meaning as an information delivery tool. The transition from this state of volatility to one of breakdown was marked by the abandonment of Komp by both actors approximately 2 months after its acquisition. Shortly after the initial interview, Haakon fell ill, which intensified his pre‐existing aversion to Komp and left it persistently turned off. With her father's end of the channel closed, Berit ceased sending messages, leading to complete nonuse over the following months. Beyond an example of domestication breakdown, the case of dyad seven illustrates the centrality of coordinated interactor efforts within the sociotechnical network to create stability. Not least due to Komp's technological make‐up, that is, a system comprising dual artefacts, dependencies emerge among actors using these: If Haakon's perception of Komp leads to rejection, Berit's ability to sustain its established meaning is compromised.

Ultimately, Komp remained wild, and the process of domestication would have to start anew. Determined to reinstate Komp's value as an information tool, Berit felt confident in her ability to motivate her father to resume his use, remarking that she was certain he wanted to keep Komp. During the last interview, Haakon did indeed revert from his initially expressed wish to return Komp but revealed that this reversion hinged on complying with his daughters' wishes:

If it’s not any better for the daughters‐ it’s really for the daughters, right? And… They probably haven’t‐ but that’s because I don’t turn it on. Because then they won’t be able to use it, if I don’t turn it on.

Although declaring to re‐attempt the domestication process, Haakon maintained that Komp was of no use to him and that its presence in his home mattered little, as he would soon die anyway.

Dyad 7's domestication breakdown was characterised by lack of synergy in negotiating constructed meanings to create stability, eventually leading to abandonment. Breakdown, however, can also involve collective rejection. Alva (early eighties) was sceptical of Komp from the moment she was offered it. Her daughter, Mari (late fifties), had already ceded her attempts to convince Alva to accept when a visiting project coordinator succeeded, leaving the Komp screen at her flat. Although having been let out of its box temporarily, Komp's process of appropriation was short‐lived, as the dyad quickly objected to its scripted design. Several aspects of Komp's script conflicted with Alva's values; its distribution of responsibilities unto relatives to invite members, initiate video calls and post pictures (including controlling their duration) left Alva feeling infantilised by the script's imposition of passivity. Consequently, she constructed an imaginary of Komp as ‘for demented people. Or Alzheimer's and stuff like that. And I don't have any of that’. Past experiences of infantilisation in her personal life, regarding her chronic condition, and in interactions with healthcare workers fuelled her opposition to the role of recipient lacking self‐efficacy. Komp's design, intended to offer relief, made Alva feel ‘like being put in prison’ by substituting a position of agency with one of anticipation. Being wheelchair‐bound, this substitution was literal, as her mobility inhibited her from reaching Komp to manually turn it off before the end of the ten‐second countdown, leaving her no option to initiate nor reject calls.

Moreover, Komp's rendition as an object of nondesire also stemmed from Alva's pre‐existing communication practices, which aligned better with her expressed values: Agency and self‐efficacy were enacted by initiating phone calls and by being able to FaceTime and save incoming pictures and videos in albums. Agreeing with her mother's objections, Mari found her own prescribed role of administrator potentially invasive and therefore problematic. Remarking that Komp's logic fundamentally conflicted with her own values, she would not wish Komp upon herself when she got older. These negotiations reveal how Komp constitutes a technology so tightly scripted that actors' space for interpreting alternative uses turns narrow if the fundamental premises of the script are not accepted, that is, the inscribed sender‐receiver role figuration. Even the previously described rescripting practices of Dyad 9, who achieved stability, and Dyad 5, who did not, relied on accepting the implications of such premises. For Dyad 12, however, Komp's script inhibited any further establishing of meaning and use, resulting in a collective rejection at the stage of appropriation.

## 4 Discussion

This study explored how older adults and their relatives domesticated the social technology Komp. The themes of stability, volatility and breakdown depict three states of different domestication trajectories, each comprising distinct constellations of actors' expectations, values, meanings and practices—some harmonious, others discordant. By revealing these trajectories of human–technology relations, this study presents both an empirically grounded reappropriation and conceptual development of the domestication framework.

### 4.1 Domestication as Stabilisation

In his conceptualisation of domestication as the ‘movement of objects into and within existing sociotechnical arrangements’, Sørensen casts this as a simultaneous process of change in the arrangement wherein technologies may ‘become stabilized’ through actors' enactments (2006, p. 47–48). Though seminal, this notion of *stabilisation* was largely employed in contemporary cultural analyses tracing technologies' trajectories on a societal level. For example, investigations on the domestication of cars (Sørensen 2006) and personal computers (Aune 1996) in Norway refer to the stability of macro‐level networks comprising sectors, communities and households. Our study is the first to reappropriate the notion of stability to inform an empirically grounded micro‐sociological account of the small‐scale family network. Recent empirical studies have reinvigorated a networked theorisation of domestication as multisited, social and collaborative enactments (Johannessen et al. 2023; Søraa et al. 2021; Lüchau and Grønning 2022). Still, although Sørensen's (2006) three‐dimensional rendition of domestication has been applied in numerous investigations of human–technology relations (e.g., Søraa and Fostervold 2021; Kleiven et al. 2020), his notion of stabilisation has been left undertheorised.

Our contribution develops the domestication framework along this axis but foregrounds states of (in)stability, with these remaining temporally sensitive and subject to change as actors reconfigure the sociotechnical constellation. Two theoretical implications can be gleaned from this development. First, it combats a depiction of domestication as a process reaching a static plateau of success, as perpetuated by some scholars (cf. Søraa et al. 2021; Airola and Rasi 2020). Rather, for domestication to happen at all, constellations of actors must continually sustain a reciprocity of actions conducive to stable use. The case of Komp illustrates this particularly well, as it comprises a technology scripted to function through networked interconnectedness. Our attention to Komp's script thus evokes the ‘essential tension between the technological and the social. […] Neither party is stable in this, though both […] seek stability’ (Silverstone 2006, 234). This tension casts stable constellations as only presently stable and as *dynamic in principle*, as the need for new negotiations may be incited by users, designers or other technologies. Such a conceptualisation sensitises domestication analyses to the transitions in capacities, needs and wishes often experienced by older adults, treating these as agents *of* and *in change* (Peine et al. 2022). Simultaneously, the attentiveness to actors beyond the end‐user provides analytical symmetry to the favouring of older adults' relations with technology by socio‐gerontechnological research (Peine and Neven 2021, 2848) and some domestication studies (e.g., Nimrod and Edan 2022; Airola and Rasi 2020). Domestication only rarely occurs in isolation (Sørensen 2006, 47), as reflected in a recent systematic review detailing the involvement of various others in older adults' incorporation of communication technology (Bavngaard et al. 2024). A networked perspective thus provides a valuable lens for studying the dynamics between (geron)technology and its various users.

The second implication concerns the constituents of stable, volatile and broken‐down states of domestication. Each is a product of tasks thought central to domestication: users' construction of practices and meanings involving the technology (Sørensen 2006; Silverstone and Hirsch 1994). What the case of Komp—and our theorisation of it—offers in this regard is an attentiveness to the potential *plurality* of intentionality across a constellation of networked actors. As our informants negotiated practices and meanings for Komp within the family constellation, the alignment of these proved not to be a prerequisite for stability; rather, harmony was. Several families incorporated Komp with differing intentions while sustaining stability through harmonious practices and meanings. In Dyad 6, Maude's enjoyment of Komp as a social tool harmonised with her daughter's consideration of Komp as providing reassurance, even despite their nonalignment. Conversely, aligned uses and meanings for Komp sometimes led to volatility or even breakdown by virtue of their internal discordance. In Dyad 10, Anna and Lotte shared their wish for Komp to be an open door to Anna but failed to establish practices harmonious with this co‐constructed meaning—mainly due to nonconformity with Komp's script. In sum, we suggest that networked actors' disparate practices and meanings can be either harmonious, creating stability or disharmonious, creating volatility and—potentially—breakdown.

### 4.2 Producing the effect(s) of Komp

The view of ‘effect’ as a predictable outcome of technological capacity has been challenged by extensive sociological scholarship (e.g., Pols and Willems 2011; Bijker and Law 1994; Orlikowski 1991). Studies investigating robots (Søraa and Fostervold 2021), apps (Neves et al. 2018) and pendant alarms (Lynch et al. 2022) have showcased—with and without the vocabulary of domestication—how incorporating such technologies involves negotiations and sometimes tensions that complicate the derivation of their health‐related ‘effect’ as intended by their designers and, variously, their users. Our contribution lies in the translation of these negotiations and tensions to states of (in)stability, sustained by a constellation of actors acting in varying degrees of harmony. The notion of effect, then, must submit to the aforementioned preconditions governing domestication: dynamicity and plurality.

If domestication involves attempts at stabilisation within dynamic constellations, as argued here, effect is less an outcome found at the end of a domestication trajectory than a contingent and continuous consequence of actors' relations at particular moments in time. Our study nuances the argument by Johannessen et al. (2023) that successfully domesticated technologies become, in the words of Silverstone and Hirsch (1994), ‘sources of power and sustenance’ for their users. This juxtaposition of successfully domesticating technology with deriving its effect risks downplaying the entanglement of effect with trajectories of use as they unfold. Pols and Willems (2011) demonstrate how attempts at defining and evaluating technology’s effect are challenged by its open and emergent nature. Our findings build upon this argument, suggesting that effect is dynamic and always *in production* through continuous tinkering in an ongoing process of domestication marked by ever‐changing states of stability. Viewed through our lens of stability‐in‐constellations, domestication *is* an effect. What sustenance is produced by and for users is a result of the types of use that a particular state of domestication permits.

We here address effect in two interrelated senses. First, as the changes in users' lives derived from using Komp in distinct ways. Second, as the distinct types of use premised by the dynamic process of domestication. This second sense denotes an effect producing an effect in the first sense. Analysing domestication processes, then, is a way of explicating observed or reported effects of Komp on users' lives, aware that these may dissolve as the constellation sustaining them reconfigures in either harmonious or discordant ways. For this pursuit, stability matters. Akrich (1994, 222) argues that only when the ‘network of technical objects and (human and nonhuman) actors is stabilised’ through negotiations involving an object's script is the effect rendered an outcome evaluable by scientific disciplines. Translating this to our analysis implies that Komp's functioning ‘in stable situations’ (Akrich 1994, 222) allows for explicating a central effect envisioned by its designers: alleviating loneliness. This implication captures well the changes experienced by Komp's intended end‐users: In all six dyads achieving stability—either through script acceptance or rescripting—older adults reported improved social connectedness, and five described how Komp alleviated feelings of loneliness. Only one such report occurred among the four older adults in volatile constellations, and none among the two whose domestication broke down.

Beyond dynamicity, we argue here for a plurality of effects. Although Komp's script prescribed alleviating loneliness as its primary goal, its interpretative flexibility (Bijker and Law 1994) afforded the production of additional effects. In the six dyads achieving stability, relatives leveraged Komp as a tool of safety and convenience: Some used video calls to assess the older adult's health condition, including whether they had fallen. Others used Komp to relieve them of the need to travel to their parents as often. Even with Komp's inscribed asymmetrical roles of active relatives and passive older adults acting as an age script (Neven 2015), the derivation of effects beyond alleviating loneliness was not a zero‐sum game wherein relatives left their older family member at home, awaiting sociality. None of the older adults wished for Komp to have bidirectional communication capabilities. Those able to use phones called their relatives to request video calls on Komp or chat about its pictures, whereas those unable to were relieved that no actions were required of them for interacting with the screen. All actors tinkered with Komp to produce their desired effects, contending with its script, other technologies in the household ecology and—importantly—each other. In describing this tinkering, we approached Komp's capacity to shift or reify intrafamily power dynamics through its implications for dyads' domestication rather than as the focus of our study. For further discussion on power dynamics surrounding Komp and on the reductiveness of a zero‐sum view of older adults' value derivation from similar technologies, see Rasmussen et al. (2021) and Berridge and Wetle (2020), respectively.

## 5 Concluding Remarks

Although we stand by Akrich's (1994) assertion that stability and effect are best discerned with the benefit of hindsight, our study shows that both are dynamic and open to change. This dynamicity carries methodological implications. Our contention that dyads' domestication has not reached closure is reflected back at us, as our analysis has not done so itself. Despite a longitudinal design, our data production forms a small incision point, conveying a story interpreted from the perspective of certain key actors at certain points in time. The dyads' trajectories after the concluding interviews remain unknown. In questioning the permanency of stability, we find it neither viable nor our ambition to portray our results as prescriptive, providing a roadmap detailing what steps to trace to consistently arrive at a stable use of technologies such as Komp. Rather, our study contributes by complementing quantifiable parameters for evaluating social technology as a tool for increased social connectedness (Akhtar 2024). It does so through its focus on constellations and negotiations, foregrounding the context‐dependent sociotechnical dynamics forming the otherwise opaque backdrop of Komp's effect(s).

## Author Contributions

**Erik Børve Rasmussen:** conceptualization, data curation, formal analysis, project administration, writing – review and editing, writing – original draft, funding acquisition. **Anne Lund:** conceptualization, data curation, formal analysis, investigation, project administration, writing – original draft, writing – review and editing. **Martin Vinther Bavngaard:** conceptualization, data curation, formal analysis, investigation, project administration, writing – original draft, writing – review and editing.

## Funding

This study is part of a collaborative research and development project that centres on communication technology in Oslo municipality, funded by the Research Council of Norway (Grant No. 331810).

## Ethics Statement

This study obtained recommendations from the Norwegian Agency for Shared Services in Education and Research (no. 589119) and the Regional Committees for Medical and Health Research Ethics (no. 516796). As detailed in the paper's section on ethics, additional steps were taken by authors to manage ethical dilemmas emerging during data collection.

## Conflicts of Interest

The authors declare no conflicts of interest.

## Permission to Reproduce Material From Other Sources

Permission to reproduce marketing material from Abilia as part of this study has been obtained.

## Data Availability

The data that support the findings of this study are available from the corresponding author upon reasonable request.
